# London Hospitals and Metropolitan Members

**Published:** 1888-04-07

**Authors:** 


### The Hospital

A Weekly Institutional Journal of

Science, Medicine, Nursing, and Philanthropy.

*For Hospitals, Asylums, Medical Practitioners, Students, Nurses, Families, Parishes, Congregations, and the Charitable Public.*

Vol. IV. No. 80.] [April 7, 1888.

### London Hospitals and Metropolitan Members.

Lord Randolph Churchill, like King David, is minded to "serve his generation" before he "falls on sleep." "Whatsoever his hand finds to do" he does it "with his might." He is Member of Parliament for Paddington. During the late distress he allowed himself to be placed at the head of the relief movement, and thus became member for the "out-of-works." In May he is to preside at the annual meeting of the St. Mary's Hospital Managers, and he will thus prove himself also member for the "out-of-healths." All these things are entirely as they should be.

Members of Parliament are public servants. In saying this we of course mean them no disparagement; still less do we affirm a claim for any other than voluntary service. The man who serves the largest number of his fellows—who serves them in their highest interests, who serves them longest and serves them best—is worthy of the noblest of all monuments his fellows have it in their power to raise. There is no work so great for one man as to serve all other men. This at least is of the essence of the Christian philosophy, as it is of every other philosophy which is worth the name.

Lord Randolph Churchill has made a beginning in the hospital region. In London, hospitals occupy a large place in the social economy—a relatively larger place than in most other towns. The number of people needing hospital aid is relatively greater than in other towns; the means of relief available are, at least in some parts of London, relatively more abundant and more important than in many other large centres. It is not to be expected that every Member of Parliament is familiar with these facts. It is sometimes not easy for a man who has lived but a short time, or who may not have lived at all as a resident, in his Parliamentary borough, to take the measure of the different public institutions which demand his care. In some boroughs there is no hospital; in others the hospital is the largest, the costliest, and the most important of all the organisations. In every case where a hospital exists, whether it be large or small, it should be the duty, as it is certainly the interest, of the Parliamentary representative, to make himself well acquainted with its financial position and its practical efficiency. He should also, wherever it is practicable, give it not merely "his countenance," but his intelligent study and the stimulus of his frequent presence and various aid.

The hugeness of London is one of its most serious misfortunes. It is not one community, and, what is worse, it is not many communities. It is a wilderness of houses, a desert of people; a wild waste in which the wilder and less scrupulous spirits can live in licence, undisturbed by the restraining influence of public opinion. There is no public opinion for the many. Unless a man be known to the world he may cast his morals to the winds without let or hindrance. It is the aim of men of practical wisdom to develop the sense of community. This is probably not possible in London as a whole; but it is possible in each separate Parliamentary constituency. It is worth a great and intelligent effort to bring the different districts of this densely-peopled Sahara within the moral control of public opinion as well as within the authority of the policeman and the rate-collector. A few energetic spirits have grasped and comprehended certain physical wants of the people, such as light and water; and, because it was monetarily profitable, have succeeded in bringing even the most debased and the most outlying districts within the practical daily operation of the gas and water systems. That which has been proved possible in the region of physical necessity may be shown to be not impossible in the region of general morality and well-being. Those who really desire to serve the highest interests of a great and universally influential people should make it a matter of careful and intelligent thought that every means available for that purpose be used promptly, and used to the utmost.

We affirm again, as we have often affirmed before, that the hospital of any Parliamentary borough is an institution which combines within itself the highest educational efficacies of church, school, public meeting, and town council. It is a common centre. It is an acknowledged necessity. Its administration demands the best judgment and skill, but precludes squabbling. In the conduct of its affairs there is no room and no necessity for those differences of opinion which compel cleavage and separation. Here, if nowhere else, the Member of Parliament, whatever his politics, may feel that he represents the whole of his constituents. Here, if anywhere, the religious separatist may prove that, in practice at least, he loves "the unity of the spirit and the bond of peace." Here the man whom no Church acknowledges, and who claims kindred with none, may show that whatever else he has cut himself off from, he has not excluded from his human sympathy the distressed and the sorrowful of the human race.

All these things are truisms which everybody admits and everybody proclaims; but, like the commandment to love one's neighbour as one's self, the belief is in many cases in the inverse ratio of the practice. The Member of Parliament has it in his power, to a large extent, to alter all this. We suggest to him that if he desires to unite his constituency into a community, and so to secure for it all the unspeakable advantages of oneness of interest and feeling, the hospital of the borough offers him a rallying point, a central meeting-ground, which not only has no superior, but which has no equal. In saying this we are using no special pleading. Let any given Member of Parliament sit down and think the thing out for himself. He will conclude that, even on the lowest ground of self-interest, a ground which we hate, and which we believe many other people detest and despise as heartily as ourselves—even on that ground, what is here said is worthy of consideration and action. But on the higher ground, the only ground worthy of a decent and wholesome subject of the Queen, the line of thought and effort here indicated will repay the most capable and conscientious study and work. If the highest possibilities of humanity lie in the well organised and instructed community, as they surely do, and if the hospital is an existing and most potential instrument for the development and strengthening of the highest life of the community, he cannot be either a good or a wise man who does not do all that lies in his power to use that instrument for its highest and noblest purposes. "He that knoweth to do good and doeth it not, to him it is an offence."

